# Effectiveness of emotional freedom techniques therapy in alleviating anticipatory grief for cancer patients

**DOI:** 10.1097/MD.0000000000044211

**Published:** 2025-09-05

**Authors:** Dandan Zheng, Wei Xiao, Dan Duan, Chunhua Tang, Xianghao Lin

**Affiliations:** a Xianning Medical College, Hubei University of Science and Technology, Xianning, China; b Yichang Central People’s Hospital, Zhijiang, China; c The Second Affiliated Hospital of Hubei University of Science and Technology, Xianning, China.

**Keywords:** acupoint tapping, anticipatory grief, cancer, emotional freedom techniques, psychosocial intervention

## Abstract

**Background:**

Cancer patients frequently experience anticipatory grief (AG), anxiety, and sleep disturbances. This randomized controlled trial evaluates the efficacy of the emotional freedom technique (EFT) therapy in alleviating these symptoms.

**Methods:**

A total of 58 cancer patients were randomly assigned to an intervention group (n = 30) receiving 4-week EFT therapy (acupoint tapping + scripted prompts, 5 minutes per prompt) plus routine care, or a control group (n = 28) receiving routine care alone. AG was measured using the Preparatory Grief in Advanced Cancer Patients scale, anxiety with the Hamilton Anxiety Rating Scale, and sleep quality via the Pittsburgh Sleep Quality Index at baseline and postintervention.

**Results:**

After 4 weeks, the intervention group demonstrated: Significantly lower AG scores versus control (*P* < .01); greater anxiety reduction (*P* = .04); improved sleep quality (*P* < .01).

**Conclusion:**

EFT therapy produces clinically significant improvements in AG, anxiety symptoms, and sleep quality among cancer patients within 4 weeks.

## 1. Introduction

Cancer is one of the most significant global health challenges.^[1]^Despite the potential of computer-assisted^[2]^ decision-making systems^[3]^ to aid in cancer diagnosis,^[4]^ the global incidence continues to rise, with an estimated 27 million new cases projected by 2040 and nearly 10 million annual deaths, meaning that approximately 1 in 8 male and 1 in 11 females will die from cancer.^[5]^ New cases and deaths for 36 cancers and all cancers combined in 2020, female breast rank 1, Lung and Prostate rank 2 and 3.^[6]^ In China, cancer constitutes a major public health burden,^[7]^ with lung, liver, stomach, colorectal, and esophageal cancers collectively accounting for >67.5% of cancer-related deaths, underscoring the urgent need for integrated biopsychosocial care.^[8]^ Beyond its physical burden, cancer significantly disrupts the psychological well-being.^[9]^ A central factor in psychological distress is illness uncertainty, defined as a cognitive state characterized by unpredictability, ambiguity, and a lack of clarity regarding illness-related events.^[10]^ Illness uncertainty is strongly correlated with anticipatory grief (AG),^[11]^ a psychological phenomenon in which patients begin mourning expected future losses, including deterioration of health, autonomy, and ultimately life.^[12]^

The concept of AG was first introduced by Lindemann in 1944, who described grief reactions occurring before an actual loss, focusing primarily on the emotional and physical symptoms experienced by individuals anticipating the death of a loved one.^[13]^ His model laid the foundation for understanding AG as a reaction to impending loss, but did not provide a structured framework for its stages or coping mechanisms. Rando model (1986), however, expanded this by conceptualizing AG as a dynamic, multifaceted process.^[14]^ She proposed that AG encompasses mourning, coping, planning, and psychological adaptation to impending losses. Rando model is more comprehensive, detailing specific stages of grief, and emphasizes that AG is both a clinical symptom and a psychological process, which allows for a more nuanced understanding of an individual’s emotional and coping responses.^[15]^ Owing to its depth and practical application, Rando framework is widely used in clinical settings, especially in guiding care for individuals facing imminent loss.^[16]^ Recent empirical studies have highlighted that AG, if left untreated, can significantly impair treatment adherence, decision-making, and emotional regulation, and ultimately contribute to poorer health outcomes, including the risk of developing prolonged grief disorder.^[17]^ A systematic review by Nielsen further underscored that AG is a distinct clinical phenomenon with unique trajectories and outcomes compared to conventional bereavement, demanding targeted intervention approaches.^[18]^

Given these challenges, the identification of effective psychosocial interventions for alleviating AG among patients with cancer is of paramount importance. emotional freedom techniques (EFT) have emerged as a promising therapeutic modality in this context.^[19]^ Developed by Gary Craig, EFT integrates the principles of meridian theory of traditional Chinese medicine with Western cognitive-behavioral strategies.^[20]^ The intervention involved tapping on specific acupuncture points while simultaneously engaging in cognitive reframing and emotional acceptance techniques. Accumulating empirical evidence supports the efficacy of EFT in reducing psychological distress across diverse populations and clinical settings.^[21]^ A systematic review found that clinical EFT is efficacious in a range of psychological and physiological conditions.^[22]^ Meta-analyses have consistently demonstrated the effectiveness of EFT in alleviating anxiety^[21]^, depression,^[23]^ and trauma-related symptoms.^[24,25]^ For example, a meta-analysis reported moderate to large effect sizes for EFT in reducing anxiety symptoms,^[26]^ whereas Sebastian found substantial improvements in PTSD and depressive symptoms following EFT interventions.^[24]^ Additionally, EFT has been successfully applied to manage stress among individuals with chronic diseases such as diabetes^[27]^ and Parkinson disease,^[28]^ and in perioperative settings to reduce surgical anxiety.^[29]^ Furthermore, the efficacy of EFT has been documented across diverse populations, including patients,^[30]^ students,^[31]^ pregnant women,^[32]^ adolescents,^[33,34]^ and individuals with obesity.^[35]^

Despite the growing body of evidence supporting EFT’s effectiveness of EFT across various mental health conditions, few studies have specifically examined its application in mitigating AG among patients with cancer. To address this critical research gap, the present study aimed to evaluate the effectiveness of EFT in reducing AG, with the ultimate goal of contributing to the development of scalable, noninvasive interventions to enhance psychological resilience in this vulnerable population.

## 2. Subject and methodology

### 2.1. Study population

The sample size was calculated using the following formula: *n* = 2(*u*α+*u*β)2σ2/δ2, where *n* is the required number of participants in each group. Considering a significance level α = 0.05, and a power β = 0.10, the critical values are *u* 0.05/2 = 1.96 and *u* 0.10 = 1.282. Referring to the study by Wang,^[36]^ this calculation yielded a sample size of 26 participants per group. The sample size was increased by 15% to account for potential dropouts and other contingencies, resulting in a final sample size of 30 participants per group. Thus, 60 cancer patients were recruited using convenience sampling from the Oncology Department of Zhijiang People’s Hospital between January and April 2024. The study employed a concurrent controlled trial design, with participants assigned to the intervention group (*n* = 30) or the control group (*n* = 30) according to their ward. All participants provided informed consent before their inclusion in the study.

#### 2.1.1. Inclusion criteria

1. Pathologically confirmed diagnosis of cancer with an expected survival of ≥6 months.
2. Age ≥ 18 years.
3. Voluntary participation with signed informed consent.
4. Karnofsky performance status (KPS) score ≥ 60.
5. Ability to read and communicate effectively.

#### 2.1.2. Exclusion criteria

1. Presence of mental illness or severe organic diseases.
2. Muscle strength score of both upper limbs < 3.
3. Participation in other psychological intervention studies prior to or during the study period.

### 2.2. Intervention methods

#### 2.2.1. Control group

The control group received standard nursing care that included comprehensive patient education and support. The nurses provided individualized in-person guidance on dietary management, medication adherence protocols, and evidence-based health education. These interventions were delivered during care routines and scheduled treatment sessions, with contact time carefully matched to the intervention group to control for potential attentional bias effects.

#### 2.2.2. Intervention group

Of the 102 patients screened, 60 were enrolled in the study. The intervention group received standard nursing care supplemented with EFT delivered by a licensed nurse with dual certification in psychotherapy and EFT specialization (TCH). Frequency and duration, including daily sessions (7 sessions/week) over 4 weeks, with each session lasting 8 to 10 minutes. Interventions continued until the subjective units of the distress scale (rated on a 0–10 scale) stabilized at ≤2 for 2 consecutive sessions, ensuring clinically meaningful distress reduction.

**Figure 1.**
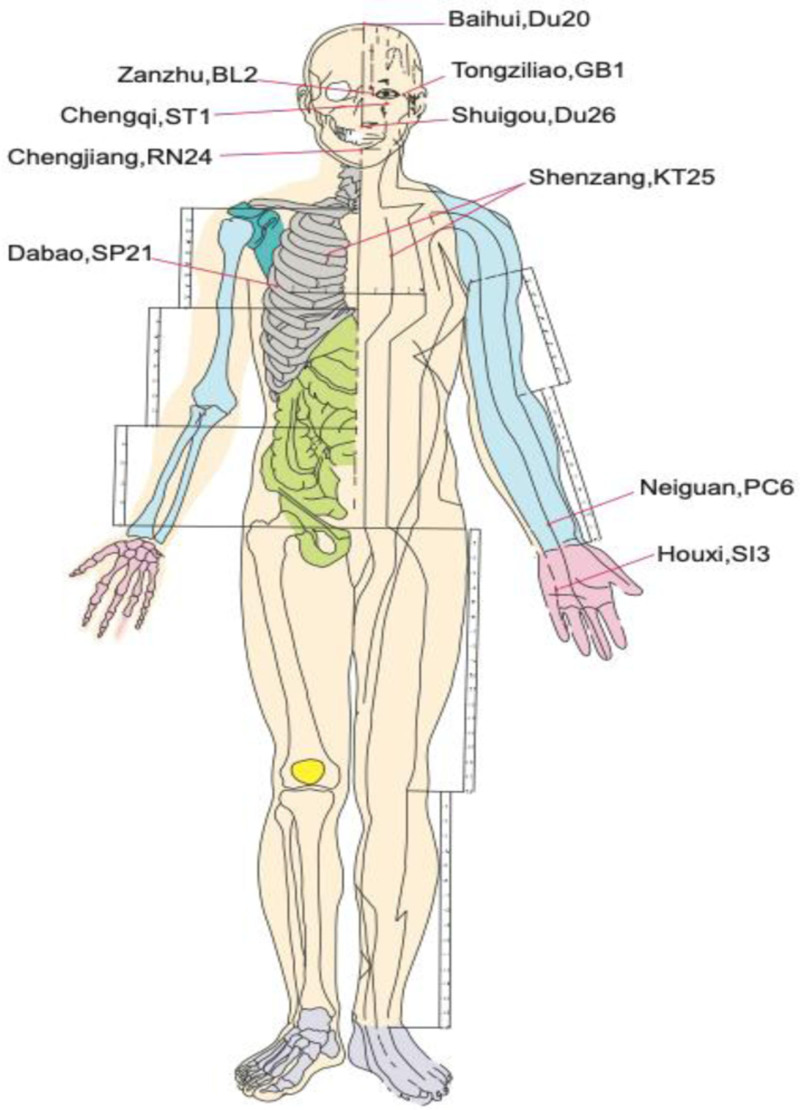
Schematic diagram of acupuncture points. This diagram shows the location of acupuncture points on the human body. The diagram presents the 10 acupuncture points of the EFT in an intuitive manner and shows their locations on the human body and the relevant anatomical landmarks. This diagram can be used as a reference to understand the spatial relationship between acupuncture points. EFT = emotional freedom techniques.

(1) Preparation phase
  1) Formation of the EFT intervention team: The team comprised 1 psychotherapist with expertise in EFT, 2 oncology nurses, and 2 postgraduate nursing students, all of whom had completed EFT theoretical training and received specialized instruction in EFT.
  2) A comfortable, quiet room was designated for the intervention sessions.
  3) Baseline assessment: Patients completed the Chinese version of the preparatory grief in advanced cancer patients (PGAC) questionnaire and a general information questionnaire to establish their personal records. The Hamilton anxiety scale and the Pittsburgh sleep quality index (PSQI) were used to assess anxiety and sleep quality, respectively.
(2) Intervention phase
1) Pre-session preparation: The patients were instructed to trim their nails, practice hand hygiene, and relax. Appropriate social distancing measures were maintained and all necessary precautions were followed.
2) Identification of the target emotion: Patients were guided to recognize and focus on negative emotions. The intensity of these emotions was evaluated using a scale.
3) Acupoints and cue phrases: Patients were instructed to tap specific acupoints (Fig. 1), including Houxi (SI3), Neiguan (PC6), Zanzhu (BL2), Tongziliao (GB1), Chengqi (ST1), Shuigou (Du26), Chengjiang (RN24), Shenzang (KT25), Dabao (SP21), and Baihui (Du20). A specific cue phrase was used: “Although I am feeling ____ (e.g., afraid) when thinking about this event, I fully and unconditionally accept myself.”
4) Tapping and cue repetition: Patients were instructed to tap the specified acupoints using their index, middle, and ring fingers with sufficient pressure to create a mild sore sensation. As they tapped, the patients repeated the cue phrase, sequentially addressing the 10 acupoints (approximately 2 minutes, with 10–12 seconds per phrase).
5) Post-tapping adjustment: After completing the tapping sequence, the patients took 3 deep breaths to expel negative emotions.
6) Consolidation and adjustment: The effect of emotional adjustment was assessed after the first round of EFT. If residual negative emotions persisted, the cue was modified to “Although I still feel a little ___ (e.g., scared) when thinking about it, I fully accept myself.” The intensity of emotions was graded and additional rounds were performed as needed, following the same process as in the first round. The intervention was continued until the subjective units of the distress scale were reduced to ≤2.
(3) Closing phase
A nurse was responsible for archiving data and images from both groups. Additionally, a WeChat group was established as the intervention group. The recorded data were systematically saved in chronological order and organized according to each round of the intervention.

### 2.3. Evaluation methods

#### 2.3.1. Evaluation indicators

Subjective units of distress scale (SUDS): This scale ranges from 0 to 10, with a score of 0 indicating no distress and a score of 10 indicating extreme and unbearable distress. The SUDS was administered both before and after the EFT intervention to provide a quick and subjective assessment of the patient’s emotional intensity. The procedure was concluded when the emotional distress level was deemed sufficiently reduced, based on the SUDS score.

PGAC: The PGAC scale, adapted from the Chinese version developed by Dajun Xin, consists of 31 items covering 7 dimensions: sadness and anger (items 3–12, 19–20), attitude towards death (items 13, 17, 23, 25), somatic symptoms (items 24, 26, 27, 29), religious comfort (items 14, 15, 31), perceived social support (items 2, 30), personal wishes (items 21, 22), and self-awareness (items 16, 18, 28). Each item is rated on a 4-point Likert scale, ranging from “disagree” (0) to “agree” (3), with higher scores indicating more severe grief responses. Cronbach’s alpha for the overall PGAC scale was 0.919, and for the individual factors, it ranged from 0.533 to 0.926. The content validity index was reported to be 0.916.

The Hamilton anxiety rating scale (HAM-A) was used to evaluate the severity of the anxiety symptoms, with a total score ranging from 0 to 56. Higher scores reflect greater anxiety severity. The scoring criteria were as follows: 0 to 17, no anxiety or mild anxiety; 18 to 24, moderate anxiety; 25 to 30, significant anxiety; and ≥31, severe anxiety.

The PSQI assesses sleep quality, with total scores ranging from 0 to 21. Higher scores indicated poorer sleep quality. The scoring criteria were as follows: 0 to 5, good sleep quality; 6 to 10, average sleep quality; 11 to 15, poor sleep quality; and 16 to 21, very poor sleep quality.

#### 2.3.2. Data collection method

The participants were assessed using a general information questionnaire and the PGAC scale at baseline by 2 trained specialist nurses. A second assessment was conducted 4 weeks later. For participants in the intervention group who were discharged during the study period, daily follow-up was conducted by an investigator through the WeChat group. The participants were required to report their progress via a check-in system each day following the intervention. The second round of data collection was completed at the end of the study period either through WeChat or by scheduling a telephone appointment.

### 2.4. Ethics

The study was approved by the Ethics Committee of Zhijiang People’s Hospital January (April 2024). (Approval No. 202440). Informed consent was obtained from all participants prior to their inclusion in the study in accordance with ethical guidelines.

### 2.5. Statistical methods

Data entry was verified by two independent researchers. Statistical analyses were performed using the SPSS version 23.0 Release 2015 (International Business Machines Corporation [IBM], Armonk). Normally distributed continuous data were presented as mean ± standard deviation (M ± SD). Non-normally distributed data were summarized using the median (P25, P75). Categorical variables are described as frequencies and percentages. A 2-sample *t*-test or chi-square test was used to compare the differences between groups, with scale scores compared using a 2-sample *t*-test. Statistical significance was set at *P* < .05.

## 3. Results

### 3.1. Comparison of general information between the 2 groups

**Figure 2.**
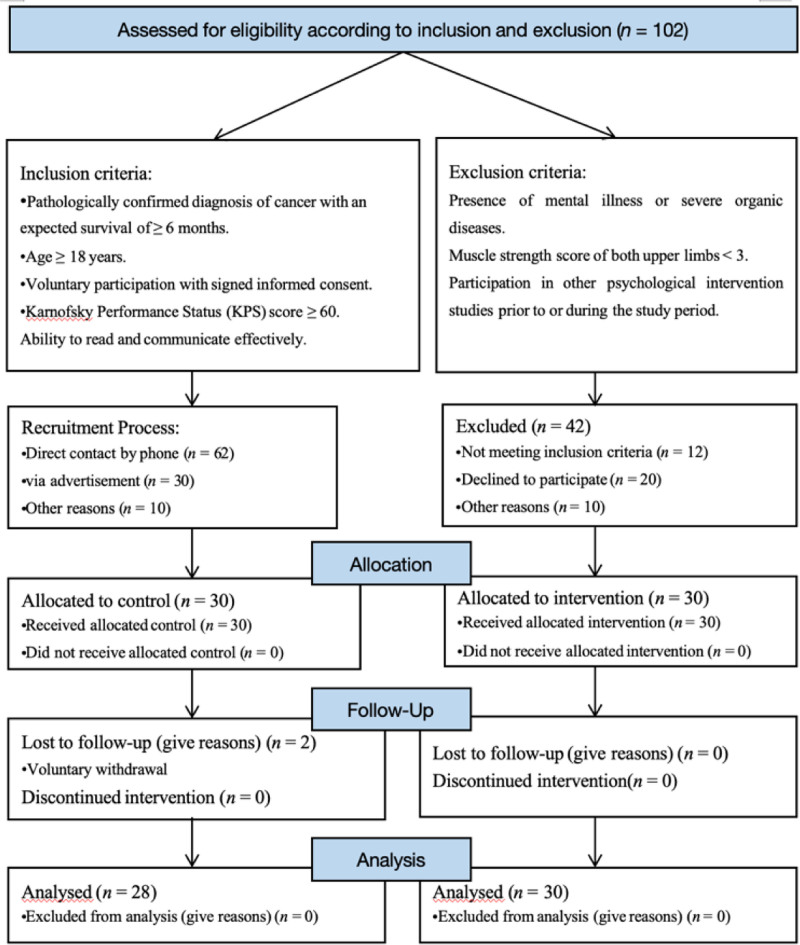
Participant recruitment and retention flowchart. This figure presents a flowchart illustrating the recruitment and retention process of study participants. It outlines the steps taken from initial participant screening to enrollment, followed by follow-up procedures, including any attrition during the study. The flowchart clearly shows the number of participants at each stage, such as those eligible, excluded, enrolled, and retained, as well as the reasons for exclusion or dropout. This figure is intended to provide a transparent view of participant progression throughout the study.

Following the 4-week intervention, 30 participants in the intervention group successfully completed the program. In the control group, 2 participants were lost to follow-up because of their inability to contact them, leaving 28 participants for the final analysis. A detailed flow of the study process is shown in Figure 2. A comparison of baseline characteristics between the 2 groups is presented in Table 1.

**Table 1:** Comparison of general information between the 2 groups.

Items	Category	Control group (*n* = 28)	Intervention group (*n* = 30)	Statistics	*P*
Gender	Male	17 (60.7)	16 (53.3)	*x*^2^ = 0.322	.571
	Female	11 (39.3)	14 (46.7)		
Age	<45 yr old	4 (14.3)	4 (13.3)	*x*^2^ =0.064	.968
	45–60 yr old	14 (50)	16 (53.3)		
	>60 yr old	10 (35.7)	10 (33.4)		
Place of residence	Rural	19 (67.9)	15 (50)	*x*^2^ = 1.904	.168
	Urban	9 (32.1)	15 (50)		
Education level	Below junior school	19 (67.9)	23 (76.7)	*Z* = 0.697	.486
	High school	7 (25)	5 (16.7)		
	High school or above	2 (7.1)	2 (6.6)		
Marital Status	Unmarried	0 (0)	0 (0)	...	1.000*
	Married	28 (100)	29 (96.7)		
	Divorced or widowed	0 (0)	1 (3.3)		
Religious belief	Yes	0 (0)	2 (6.6)	...	.503*
	No	28 (100)	28 (93.4)		
TNM Staging	Phase I	0 (0)	0 (0)	*Z* = 0.155	.877
	Phase II	6 (21.4)	3 (10)		
	Phase III	15 (53.6)	22 (73.3)		
	Phase IV	7 (25)	5 (16.7)		
Course of disease	<1 mo	1 (3.6)	3 (10)	*Z* = 0.162	.871
	1–7 mo	16 (57.1)	14 (46.7)		
	7–12 mo	4 (14.3)	3 (10)		
	>12 mo	7 (25)	10 (33.3)		
Work status	Employed	1 (3.6)	3 (10)	*x*^2^ = 0.978	.613
	Unemployed	16 (57.1)	16 (53.3)		
	Retired	11 (39.3)	11 (36.7)		
Medical payment methods	NCMS	11 (39.3)	13 (43.3)	*x*^2^ =0.342	.952
	Employees’ health insurance	3 (10.7)	3 (10)		
	Resident Health Insurance	12 (42.9)	11 (36.7)		
	Self-funded	2 (7.1)	3 (10)		
Number of cases	Lung cancer	9 (32.1)	11 (36.7)	*x*^2^ =0.131	.988*
	Breast cancer	4 (14.3)	4 (13.3)		
	Bowel cancer	2 (7.1)	2 (6.7)		
	Nasopharyngeal esophageal cancer	13 (46.4)	13 (43.3)		
First hospitalization	Yes	2 (7.1)	3 (10)	*x*^2^ =0.000	1.000*
	No	26 (92.9)	27 (90)		
Treatment modality	Radiotherapy and chemotherapy	20 (71.4)	21 (70)	*x*^2^ =0.289	.865
	Radiotherapy	1 (3.6)	2 (6.6)		
	Chemotherapy	7 (25)	7 (23.4)		

This table presents a comparison of the general demographic and baseline characteristics of the 2 groups. Variables such as age, sex, education level, and other relevant factors were compared to ensure equivalence between the groups before the intervention. No statistically significant differences were observed in the general characteristics.

NCMS = new rural cooperative medical system.

* Fisher exact probability method.

### 3.2. AG results

Table 2 provides a comparison of AG scores between the intervention and control groups, before and after the intervention. The differences between the pre- and post-intervention scores were statistically significant (*P* < .05, *P* < .01, respectively).

**Table 2:** Comparison of anticipatory grief (AG) scores between the 2 groups before and after the intervention.

Groups	Timing	Sadness and anger	Attitude towards death	Somatic symptoms	Religious comfort	Perceived social support	Disease adjustment	Self-awareness	Overall score
Control group (*n* = 28)	Pre-intervention	19.32 ± 2.19	6.14 ± 1.30	6.00 ± 1.49	4.57 ± 1.42	5.14 ± 1.53	5.36 ± 1.28	8.32 ± 0.94	54.86 ± 4.16
	Post-intervention	20.29 ± 2.57	7.11 ± 1.71	5.50 ± 1.60	4.61 ± 1.62	5.43 ± 1.40	5.18 ± 1.49	8.54 ± 0.88	55.70 ± 6.53
Intervention group (*n* = 30)	Pre-intervention	19.40 ± 4.63	6.47 ± 1.74	5.90 ± 2.12	5.50 ± 1.48	5.77 ± 1.87	4.70 ± 1.56	8.37 ± 1.50	55.75 ± 4.65
	Post-intervention	9.97 ± 3.25	4.00 ± 1.62	3.30 ± 1.68	3.67 ± 1.83	3.40 ± 1.28	4.10 ± 1.90	8.10 ± 2.12	36.53 ± 6.30
Control group before and after intervention		1.480	3.540*	−1.355	0.089	0.812	−0.501	0.972	2.027*
Intervention group before and after intervention		15.473**	8.606**	14.704**	8.137**	9.091**	2.226**	1.052	23.844**
Before intervention in both groups		0.082	0.800	−0.206	2.431*	1.384	−1.747	0.137	1.293
After intervention in both groups		13.348**	7.115*	5.094**	2.070*	5.775**	2.393*	1.008	13.043**

This table shows a comparison of anticipatory grief scores between the 2 groups before and after the intervention. The scores were assessed using a standard grief measurement scale, and the results demonstrated changes in grief intensity across time in both groups. Significant reductions in anticipatory grief scores were observed in both groups after the intervention, with the intervention group showing a more substantial decrease.

AG = anticipatory grief.

* *P* < .05.

** *P* < .01.

### 3.3. PSQI results

Table 3 presents a comparison of PSQI scores between the 2 groups before and after the intervention. The data followed a normal distribution, and independent samples t-tests were conducted to compare the mean differences in continuous variables between the 2 groups’ pre- and post-intervention scores (*P* = .68, *P* < .01).

**Table 3:** Comparing the PSQI of the 2 groups before and after the intervention.

Groups	Pre-intervention	Post-intervention	*t*	*P*
Control group (*n* = 28)	16.70 ± 1.622	16.83 ± 1.533	0.422	.68
Intervention group (*n* = 30)	16.53 ± 1.432	10.33 ± 1.516	16.511	<.01
*t*	0.327	16.286		
*P*	.745	<.01		

This table compares the PSQI scores of the 2 groups before and after the intervention. The PSQI was used to assess sleep quality, and the table highlights any changes in sleep patterns and quality in both the groups. After the intervention, the intervention group exhibited a more pronounced improvement than the control group.

PSQI = Pittsburgh sleep quality index.

### 3.4. HAM-A results

Table 4 shows the comparison of HAM-A scores between the 2 groups pre- and post-intervention (*P* = .38, *P* < .05).

**Table 4:** Comparing the HAM-A of the 2 groups before and after the intervention.

Groups	Pre-intervention	Post-intervention	*t*	*P*
Control group (*n* = 28)	25.68 ± 6.43	19.40 ± 5.72	3.866	.38
Intervention group (*n* = 30)	25.80 ± 7.59	16.50 ± 5.06	5.580	.04
*t*	−0.066	16.286		
*p*	.611	.277		

This table displays the comparison of the Hamilton anxiety rating scale (HAM-A) scores between the 2 groups before and after the intervention. The HAM-A was used to measure anxiety levels, and the table presents the changes in anxiety symptoms in both groups. The intervention groups demonstrated a decrease in post-intervention anxiety levels.

HAM-A = Hamilton anxiety rating scale.

## 4. Discussion

This study demonstrated that after 4 weeks of EFT intervention, the AG scores in the intervention group were significantly lower than those in the control group. The differences between the pre- and post-intervention scores were statistically significant (*P *< .05, *P* < .01), indicating that EFT can effectively reduce AG in patients with cancer. These findings are consistent with prior research.^[37,38]^ Furthermore, the PSQI scores improved, with the intervention group showing significantly lower scores than the control group, indicating a positive impact of EFT on sleep quality. This is consistent with Tang et al^[39]^ and Kalroozi et al^[40]^ on patients with breast cancer. Additionally, HAM-A scores in the intervention group were significantly lower than those in the control group, with statistically significant differences before and after the intervention. This is the same as Ningsih’s result,^[41]^ which recommended that health providers, especially nurses, use EFT therapy as a non-pharmacological therapy to decrease anxiety.

AG often begins with cancer diagnosis, with the nature of negative emotions evolving at different stages of the disease.^[42]^ In the early stages, fear and uncertainty dominate, as psychological adjustment to diagnosis may lead to pessimism. During treatment, emotions such as anxiety, depression, anger, and grief arise, which are often related to the side effects of treatment. In later stages, fear of death and social isolation become prevalent, with some patients exhibiting suicidal tendencies.^[43]^ This study further found that AG levels increase as the disease progresses, manifesting various negative emotions at different stages. EFT effectively alleviates these negative emotions, improves sleep quality, and reduces anxiety by regulating endocrine function and lowering cortisol levels.^[44]^ Tapping acupuncture points, a key element of EFT, has been shown to reduce the size of the amygdala and decrease cortisol secretion.^[45]^ The amygdala is the brain’s fear center and is responsible for the memory of fear; however, it is also the brain’s emotional center. The body’s endocrine activity regulates emotions through the “amygdaloid-hypothalamic-pituitary” signaling axis,^[46]^ and changes in the intensity of facial expressions (from neutral to happy or fear to neutral) are combined with the participants’ ongoing bilateral amygdala activity.^[47]^ Since cortisol levels are directly linked to negative emotions, such as anxiety and depression, tapping designated acupoints can reduce cortisol secretion, unblock meridians, and promote energy flow, thereby reducing negative emotions. For instance, tapping on specific acupoints (e.g., SI3 for fatigue, PC6 for anxiety, BL2 for mental calm, and ST1 for sadness) has been shown to relieve various psychological and physiological symptoms.^[48]^

The study found no statistically significant differences in disease adjustment and self-awareness scores between or within groups before and after the intervention (Table 2). This null finding may be attributed to several factors: The relatively short intervention duration may have been insufficient to affect measurable changes in these complex psychological constructs; patients’ preexisting cognitive frameworks regarding disease adaptation and self-awareness may have remained relatively stable during the brief study period. These results suggest that disease adjustment and self-awareness may require long-term or intensive interventions to demonstrate measurable changes. Future research should incorporate qualitative patient narratives to better understand these processes, while exploring modified intervention approaches that might more effectively target these specific outcomes.

The combination of tapping and affirmational cues during EFT intervention proved particularly effective. Patients were instructed to tap acupoints while verbalizing cues such as “Although I feel [emotion], I fully accept myself,” gradually reducing the intensity of the emotion through neuro-linguistic programming. This method allows patients to confront their illnesses, release negative emotions, and achieve emotional stability. One patient in this study, who initially feared alienation from their family, expressed the following after the intervention: “During the emotional release, the tightness in my chest disappeared. I was afraid of that feeling before, but now I feel at peace.” Another middle-aged patient stated, “I am at the prime age to work, but my illness has made me unable to support my family. This made me very sad, but after the therapy, the sadness eased.” These responses illustrate that EFT can effectively relieve negative emotions and anxiety, providing patients with emotional relief and improving their quality of life. Furthermore, some studies found that EFT can effectively reduce the cost of hospitalized patients,^[49]^ but specific studies on EFT’s impact of EFT on health care costs for cancer patients and its economic toxicity are lacking.^[50–52]^

In summary, our findings suggest that EFT has potential clinical applications in alleviating AG and improving sleep quality in patients with cancer. By reducing anxiety and enhancing sleep, EFT provides a non-pharmacological treatment option that can help cancer patients cope better with the psychological and physical stress associated with the disease. However, despite these promising results, further research is needed to assess the long-term effects and safety of EFT as well as to explore the underlying mechanisms. Future studies should also investigate the efficacy of EFT across different stages of cancer and individual differences, as well as its potential in combination with other therapies, to broaden its clinical applications and offer more comprehensive and personalized treatment options for cancer patients.

## 5. Conclusion

Overall, this study indicates that EFT significantly reduces AG in cancer patients, with additional benefits such as improved sleep quality, reduced anxiety symptoms, and enhanced emotional stability, leading to a better quality of life. EFT is cost-free, noninvasive, easy to learn, and easy to use, making it an attractive therapeutic option. However, limitations include the small sample size and single-center study design, which may limit the generalizability of the findings. Additionally, the study only followed patients for 4 weeks, and there is a lack of long-term follow-up data. Future studies should employ more rigorous designs to validate the long-term effects and clinical applicability of EFT in the mental health management of patients with cancer.

## Author contributions

**Conceptualization:** Dandan Zheng.

**Data curation:** Wei Xiao, Dandan Zheng.

**Formal analysis:** Dandan Zheng.

**Funding acquisition:** Xianghao Lin.

**Investigation:** Chunhua Tang.

**Methodology:** Dandan Zheng, Xianghao Lin.

**Project administration:** Chunhua Tang, Xianghao Lin.

**Resources:** Dandan Zheng.

**Software:** Wei Xiao, Dandan Zheng.

**Supervision:** Dan Duan.

**Validation:** Xianghao Lin.

**Visualization:** Wei Xiao.

**Writing – original draft:** Dandan Zheng.

**Writing – review & editing:** Dan Duan.
